# Survey data of COVID-19-related knowledge, attitude, and practices among indonesian undergraduate students

**DOI:** 10.1016/j.dib.2020.105855

**Published:** 2020-06-12

**Authors:** Muhammad Saefi, Ahmad Fauzi, Evi Kristiana, Widi Cahya Adi, M. Muchson, M. Eval Setiawan, Novita Nurul Islami, Dian Eka Aprilia Fitria Ningrum, M. Alifudin Ikhsan, Mavindra Ramadhani

**Affiliations:** a Faculty of Mathematics and Natural Sciences, Universitas Negeri Malang, Indonesia; b Faculty of Teacher Training and Education, Universitas Muhammadiyah Malang, Indonesia; c Faculty of Teacher Training and Education, Universitas Islam Jember, Indonesia; d Faculty of Science and Technology, Universitas Islam Negeri Walisongo Semarang, Indonesia; e Faculty of Tarbiyah and Teacher Training, Institut Agama Islam Negeri Kerinci, Indonesia; f Faculty of Teacher Training and Education, Universitas Jember, Indonesia; g Faculty of Tarbiyah and Teacher Training, Universitas Islam Negeri Maulana Malik Ibrahim Malang, Indonesia; h Faculty of Social Sciences, Universitas Negeri Malang, Indonesia; i Faculty of Industrial and Systems Engineering, Institut Teknologi Sepuluh Nopember Surabaya, Indonesia

**Keywords:** Survey data, COVID-19 pandemic, KAP survey, Knowledge, Attitude, Practice, Indonesian, Undergraduate students

## Abstract

The dataset presented in this paper is an examination of COVID-19-related knowledge, attitude, and practice among Indonesian undergraduate students. The data were collected during first month of college or university closure due to COVID-19 through a survey distributed via an online questionnaire, assessing sociodemographic information (6 items), knowledge (18 items), attitude (6 item), and practice (12 items), from 27^th^ April and 2^nd^ May 2020, gathering a total of 6,249 responses. A combination of purposive and snowball techniques helped to select the respondents via Whatsapp from more than ten universities in Indonesia. The survey data were analyzed using descriptive and inferential statistics. The data will assist in preventing and curbing the spread of COVID-19 in the university and can assist with planning for educational interventions for students’ awareness.

Specifications Table

**Table:** 

Subject	Public health
Specific subject area	Health psychology, Social psychology
Type of data	Primary data, Tables
How data were acquired	Data was gathered using an online survey platform (google forms). The questionnaire is provided as a supplementary file
Data format	Raw, Analyzed, Filtered (descriptive and inferential statistics)
Parameters for data collection	The survey data was obtained from 6249 respondents of Indonesian undergraduate students with internet access. Only students who have department and faculty approval can access the survey.
Description of data collection	The data was conducted through an online questionnaire, which was delivered to undergraduate students in Indonesia using the combination of purposive and snowball techniques helped to select the respondents via Whatsapp.
Data source location	Region: Asia Country: Indonesia
Data accessibility	Dataset is uploaded on Mendeley Repository Name: Mendeley Direct URL to data: https://data.mendeley.com/datasets/scgh3swptb/draft?a=aeea2402-bddd-45dc-80c1-a5e70f68b4b9

## Value of the data

• The data are important because this is the first survey that involved thousands of participants. So far, this survey involved the largest number of participants that explore knowledge, attitude, and practice about COVID-19 among Indonesian undergraduate students.
• The data will be useful for reseachers who want to compare with similar studies on COVID-19 related knowledge, attitude, and practice from other countries around the world, especially among undergraduate students or contributing to meta-analysis in the future.
• The data will be valuable to reseachers who want examine relationship between sociodemographics, knowledge, attitude, and practice of COVID-19 among undegraduate students.
• The details of the analyzed data are beneficial to enhancing institutional leaders’ and policymakers's awareness of the level of students’ knowledge, attitude, and practice, so institution may better prepared for preventing and curbing the spread of COVID-19 in their environment and assist with planning for educational interventions for students’ awareness.

## 1 Data description

The data set provides an insighful information based on survey data on knowledge, attitude, and practice among Indonesian undegraduate students about COVID-19. The survey involved 6,249 Indonesian undergraduate students during first month of college or university closure due to COVID-19. The data include four major groups of variable: (A) Individual demographics, including gender, age, place of current residence, spent year in university, majors of education, and occupation. (B) 18 items measured their COVID-19 related knowledge including etiology, symptoms, risk groups, transmission, and prevention. Each question of the knowledge section was rated in such a way that a score of one was given to correct responses and a score of zero was used for incorrect. (C) Six items measured their COVID-19 related attitude including reception of information, social interaction, and self motivation. A three-point Likert scale was utilized from 1 (disagree) to 3 (agree) with a neutral midpoint (point 2). (D) 12 items measured their COVID-19 related practice including compliance, prevention efforts, and a clean and healthy lifestyle. A three-point Likert scale was utilized from 1 (never) to 3 (always). The questionnaire is provided as a supplementary file. Demographic characteristics of respondents are presented in Table 1. The detailed assessments of responses on COVID-19-related knowledge, attitude, and practice by undergraduate students of Indonesian are depicted in Table 2–4. The detailed description of relationship between sociodemographics, knowledge, attitude, and practice are depicted in Table 5–9.

**Table 1:** Sociodemographic characteristics of the participants (*n* = 6249).

Variable	Freq (n)	%
Gender		
Male	1677	26.84
Female	4572	73.16
Age		
≤20	4423	70.78
>20	1826	29.22
Place of current residence		
Cities	4184	66.95
Rural	2065	33.05
Spent year in university		
1 year	2337	37.40
2 year	1881	30.10
3 year	1302	20.84
4 year	640	10.24
5 year	89	1.42
Majors of education		
Medicines and public health	546	8.74
Science and technology (ex. Biology, Physics, Engineering etc.)	764	12.23
Socials and humanities (ex. Politics, Arts etc.)	4939	79.04
Occupations		
Students	5603	89.66
Students and workers	646	10.34

**Table 2:** Response to knowledge items (*n* = 18 items).

Questions	Correct answer		Wrong answer	
	Freq (n)	%	Freq (n)	%
K1. COVID-19 is a disease caused by coronavirus	2437	39.00	3812	61.00
K2. The main clinical symptoms of COVID-19 are fever, fatigue, dry cough, and myalgia	5606	89.71	643	10.29
K3. People with COVID-19 also show no symptoms, called OTG (People without Symptoms)	5736	91.79	513	8.21
K4. Not everyone with COVID-19 has an increasingly severe condition, except the elderly	4834	77.36	1415	22.64
K5. People with COVID-19 who have chronic diseases such as diabetes, heart disease, and obesity have an increasingly severe condition	4999	80.00	1250	20.00
K6. Children and teenagers do not need to make efforts to prevent COVID-19 infection because they have a strong immune system	5716	91.47	533	8.53
K7. People with a strong immune system will not get infected with COVID-19	2785	44.57	3464	55.43
K8. People with COVID-19 who show no symptoms or OTG (People without symptoms) cannot infect the virus to others	5013	80.22	1236	19.78
K9. COVID-19 is spread through the respiratory droplets of people infected with COVID-19	4936	78.99	1313	21.01
K10. The dead bodies of people with COVID-19 who have not been buried can be a source of the spread of the COVID-19 virus	4239	67.83	2010	32.17
K11. The buried dead bodies of people with COVID-19 can be a source of the spread of the COVID-19	4437	71.00	1812	29.00
K12. COVID-19 cannot penetrate cloth masks that are commonly worn by the public	1692	27.08	4557	72.92
K13. COVID-19 only spreads through objects, it is not airborne	2543	40.69	3706	59.31
K14. Currently, there is no effective drug for COVID-19, but the treatment of early symptoms and intensive care can help people with COVID-19 to recover	5551	88.83	698	11.17
K15. To prevent COVID-19 infection, we must avoid going to crowded places like markets and train stations as well as avoid using public transportation	6116	97.87	133	2.13
K16. Avoid travel across cities can prevent the spread of COVID-19	5979	95.68	270	4.32
K17. The transmission of the COVID-19 virus can be prevented by not touching the face	5311	84.99	938	15.01
K18. Isolation and treatment of people infected with the COVID-19 virus are effective ways to reduce the spread of the virus	6128	98.06	121	1.94

**Table 3:** Attitude toward COVID-19 infection prevention (*n* = 6 items).

Questions	Disagree		Not sure		Agree	
	Freq (n)	%	Freq (n)	%	Freq (n)	%
A1. Keeping up with the information regarding the number of COVID-19 cases is important for the community	115	1.84	260	4.16	5874	94.00
A2. After knowing the information on the number of cases of COVID-19, I felt worried/scared	200	3.20	3349	53.59	2700	43.21
A3. Keeping up with the information regarding the government's call for COVID-19 preventive efforts is important for the community	851	13.62	1640	26.24	3758	60.14
A4. All people with COVID-19 are those who violate the government's call in the efforts to prevent transmission of COVID-19	416	6.66	254	4.06	5579	89.28
A5. People with COVID-19 should not be given a negative stigma in society	97	1.55	44	0.70	6108	97.74
A6 People with COVID-19 who isolate themselves show that they have a responsibility in preventing the transmission of COVID-19	97	1.55	45	0.72	6107	97.73

**Table 4:** Practice related to COVID-19 infection prevention (*n* = 12 items).

Questions	Never		Occasionally		Always	
	Freq (n)	%	Freq (n)	%	Freq (n)	%
P1. In the last few days, have you worn a mask when you were in a crowded place?	218	3.49	601	9.62	5430	86.89
P2. In the last few days, have you implemented physical distancing when you were in the crowd?	152	2.43	849	13.59	5248	83.98
P3. In the last few days, have you used hand sanitizer when you were in crowded places?	832	13.31	1863	29.81	3554	56.87
P4. In the last few days, have you washed your hands with soap after going to a crowded place?	91	1.46	509	8.15	5649	90.40
P5. In the last few days, have you immediately changed your clothes before entering the house and having contact with family members?	478	7.65	1998	31.97	3773	60.38
P6. As a college student, have you educated people around you with the knowledge of the preventive efforts of COVID-19?	307	4.91	2257	36.12	3685	58.97
P7. In the last few days, I have eaten vegetables and fruit.	63	1.01	1180	18.88	5006	80.11
P8. In the last few days, I have had enough rest.	144	2.30	1580	25.28	4525	72.41
P9. In the last few days, I have been exercising routinely.	726	11.62	3694	59.11	1829	29.27
P10. In the last few days, I have taken vitamins or supplements to increase my immune system.	1285	20.56	2746	43.94	2218	35.49
P11. In the last few days, I have been cleaning up my house more frequently.	46	0.74	485	7.76	5718	91.50
P12. In the last few days, I have been washing my hand with soap more frequently.	27	0.43	415	6.64	5807	92.93

**Table 5:** Correlation between scores of knowledge, attitude, and practice.

Variable	R square	*p*-value
Knowledge-Attitude	0.186	0.000
Knowledge-Practice	0.040	0.000
Attitude-Practice	0.021	0.000

**Table 6:** Comparison of demographic characteristics and mean KAP scores (*n* = 6249).

Variable	Freq (n)	Knowledge score		Attitude score		Practice score	
		Mean (SD)	*p*-value	Mean (SD)	*p*-value	Mean (SD)	*p*-value
Gender							
Male	1677	13.14 (2.76)	0.000	16.56 (1.72)	0.415	31.06 (3.80)	0.000
Female	4572	13.57 (2.22)		16.53 (1.42)		31.92 (3.07)	
Age							
≤20	4423	13.43 (2.35)	0.218	16.48 (1.52)	0.000	31.65 (3.29)	0.087
>20	1826	13.51 (2.47)		16.66 (1.46)		31.80 (3.33)	
Place of current residence							
Cities	4184	13.36 (2.40)	0.000	16.48 (1.54)	0.000	31.54 (3.33)	0.000
Rural	2065	13.63 (2.34)		16.64 (1.43)		31.99 (3.23)	
Spent year in university							
1 year	2337	13.38 (2.33)	0.089	16.48 (1.50)	0.000	31.63 (3.34)	0.005
2 year	1881	13.40 (2.44)		16.46 (1.54)		31.61 (3.34)	
3 year	1302	13.55 (2.37)		16.63 (1.44)		31.74 (3.28)	
4 year	640	13.61 (2.39)		16.75 (1.45)		32.14 (2.97)	
5 year	89	13.64 (2.56)		16.70 (1.77)		31.36 (3.96)	
Majors of education							
Medicines and public health	546	13.93 (2.13)	0.000	16.72 (1.30)	0.007	32.60 (2.72)	0.000
Science and technology	764	13.62 (2.39)		16.56 (1.51)		31.582(3.13)	
Socials and Humanities	4939	13.37 (2.41)		16.51 (1.52)		31.57 (3.37)	
Occupations							
Students	5603	13.49 (2.33)	0.000	16.54 (1.47)	0.891	31.73 (3.21)	0.019
Students and workers	646	13.09 (2.78)		16.53 (1.76)		31.35 (4.00)	

## 2 Experimental design, materials and methods

The research adopted a descriptive online cross-sectional survey design to evaluate COVID-19-related knowledge, attitude, and practice among Indonesian undegraduate students. The dataset included thousands responses collected between 27^th^ April and 2^nd^ May 2020 from more than ten universities in Indonesia. Due to the universities were closed at the time of data collection, it was not feasible to conduct population-based survey. The main researchers opted to use WhatsApp Messenger for enrolling potential participants. A questionnaire was designed and executed using google forms and link generated was shared on WhatsApp groups of faculties. Link was also shared personally to other faculties. Faculties were required to complete the consent from before forwarding the URL to their students. The delivered link to undergraduate students using the combination of purposive and snowball techniques helped to select the respondents. The inclusion criteria were (1) undergraduate students, (2) healthy without COVID-19, and (3) never suffered from COVID-19. A total of 6,252 responses were received, but three responses were eliminated because were met criteria. Finally, 6,249 responses were used for further analysis.

The original items of the Questionnaire were generated from the results of literature reviews according to previous study towards COVID-19 [1], [2], [3], and MERS-SARS [4,5], and the explanation about COVID-19 informed in the WHO's website [6]. After translating to indonesian with applied the combined techniques [7], the questionnaire was sent to three infectious disease specialists at Muhammadiyah hospital to get their opinions regarding its simplicity, relevance, clarity, and comprehensive [8]. *Re*-validated using Rasch model measurement showed that the questionnaire having acceptable reliability and validity, with Real item reliability (Real RMSE) 0.97 for attitude scale, 0.98 for knowledge scale, and 0.99 for practice scale.

The individual demographics are the potential sources related students’ knowledge, attitude, and practices. The respondents’ demographics, COVID-19-related knowledge, attitude, and practice were analyzed using frequencies and percentages. Pearson's rank correlation analyses to understand the relationships between knowledge, attitude, and practice. Independent samples *t*-test and one-way ANOVA were performed in assessing any difference in mean score by demographic characteristics. Chi-square tests were applied to find difference in groups (good vs poor) by demographic characteristics. A binary logistic regression analysis was applied as odds ratio (OR) and 95% confidence interval (CI) to find possible determinants of good knowledge, attitude, and practice. The hierarchical (orsequential) multiple regression was conducted to determine whether the variance explained increased significantly with the addition of all variable. A p-value of less than 0.05 were considered significant in all tests.

## Declaration of Competing Interest

The research project did not receive financial support from any institutions. The authors declare that they have no known competing financial interests or personal relationships that could have appeared to influence the work reported in this paper.

## Appendix Supplementary materials

Image, application 1
