# Supplementary material for: Survey data of COVID-19-related knowledge, attitude, and practices among indonesian undergraduate students
Source: Data Brief. 2020 Jun 12;31:105855. doi: 10.1016/j.dib.2020.105855 (PMC7291994; doi:10.1016/j.dib.2020.105855)
Supplement: Supplementary file 1 [file mmc1.docx]

**Questionnaires for COVID-19 related KAP Survey**

This questionnaire is designed to assess the knowledge, attitude and practices of undergraduate students towards COVID-19 infection. Please take time to read and answer each question carefully by circling the best alternative that represents your response.

1. **Section A: Demographics**
2. Gender
3. Male
4. Female
5. Age
6. Less than or equal to 20 year
7. more than 20 year
8. Current Residence
9. City
10. Rural
11. Length of Study
12. 1 year
13. 2 year
14. 3 year
15. 4 year
16. 5 year
17. Major
18. Medicines and public healths
19. Sciences and technologies (ex. Biology, Physics, Engineering etc.)
20. Socials and humanities (ex. Politics, Arts etc.)
21. Occupation
22. Students
23. Students and workers
24. **Section B: Knowledge**
25. COVID-19 is a disease caused by coronavirus
26. Yes
27. No
28. The main clinical symptoms of COVID-19 are fever, fatigue, dry cough, and myalgia
29. Yes
30. No
31. People with COVID-19 also show no symptoms, called OTG (People without Symptoms)
32. Yes
33. No
34. Not everyone with COVID-19 has an increasingly severe condition, except the elderly
35. Yes
36. No
37. People with COVID-19 who have chronic diseases such as diabetes, heart disease, and obesity have an increasingly severe condition
38. Yes
39. No
40. Children and teenagers do not need to make efforts to prevent COVID-19 infection because they have a strong immune system
41. Yes
42. No
43. People with a strong immune system will not get infected with COVID-19
44. Yes
45. No
46. I don’t know
47. People with COVID-19 who show no symptoms or OTG (People without symptoms) cannot infect the virus to others
48. Yes
49. No
50. COVID-19 is spread through the respiratory droplets of people infected with COVID-19
51. Yes
52. No
53. The dead bodies of people with COVID-19 who have not been buried can be a source of the spread of the COVID-19 virus
54. Yes
55. No
56. I don’t know
57. The buried dead bodies of people with COVID-19 can be a source of the spread of the COVID-19
58. Yes
59. No
60. I don’t know
61. COVID-19 cannot penetrate cloth masks that are commonly worn by the public
62. Yes
63. No
64. COVID-19 only spreads through objects, it is not airborne
65. Yes
66. No
67. I don’t know
68. Currently, there is no effective drug for COVID-19, but the treatment of early symptoms and intensive care can help people with COVID-19 to recover
69. Yes
70. No
71. To prevent COVID-19 infection, we must avoid going to crowded places like markets and train stations as well as avoid using public transportation
72. Yes
73. No
74. Avoid travel across cities can prevent the spread of COVID-19
75. Yes
76. No
77. The transmission of the COVID-19 virus can be prevented by not touching the face
78. Yes
79. No
80. Isolation and treatment of people infected with the COVID-19 virus are effective ways to reduce the spread of the virus
81. Yes
82. No
83. **Section C: Attitudes**
84. Keeping up with the information regarding the number of COVID-19 cases is important for the community
85. Agree
86. Disagree
87. Not sure
88. After knowing the information on the number of cases of COVID-19, I felt worried/scared
89. Agree
90. Disagree
91. Not sure
92. Keeping up with the information regarding the government's call for COVID-19 preventive efforts is important for the community
93. Agree
94. Disagree
95. Not sure
96. All people with COVID-19 are those who violate the government's call in the efforts to prevent transmission of COVID-19
97. Agree
98. Disagree
99. Not sure
100. People with COVID-19 should not be given a negative stigma in society
101. Agree
102. Disagree
103. Not sure
104. People with COVID-19 who isolate themselves show that they have a responsibility in preventing the transmission of COVID-19
105. Agree
106. Disagree
107. Not sure
108. **Section D: Practices**
109. In the last few days, have you worn a mask when you were in a crowded place?
110. Always
111. Occasionally
112. Never
113. In the last few days, have you implemented physical distancing when you were in the crowd?
114. Always
115. Occasionally
116. Never
117. In the last few days, have you used hand sanitizer when you were in crowded places?
118. Always
119. Occasionally
120. Never
121. In the last few days, have you washed your hands with soap after going to a crowded place?
122. Always
123. Occasionally
124. Never
125. In the last few days, have you immediately changed your clothes before entering the house and having contact with family members?
126. Always
127. Occasionally
128. Never
129. As a college student, have you educated people around you with the knowledge of the preventive efforts of COVID-19?
130. Always
131. Occasionally
132. Never
133. In the last few days, I have eaten vegetables and fruit.
134. Always
135. Occasionally
136. Never
137. In the last few days, I have had enough rest.
138. Always
139. Occasionally
140. Never
141. In the last few days, I have been exercising routinely.
142. Always
143. Occasionally
144. Never
145. In the last few days, I have taken vitamins or supplements to increase my immune system.
146. Always
147. Occasionally
148. Never
149. In the last few days, I have been cleaning up my house more frequently.
150. Always
151. Occasionally
152. Never
153. In the last few days, I have been washing my hand with soap more frequently.
154. Always
155. Occasionally
156. Never
